# Looking to the Future

**Published:** 1919-04

**Authors:** 


### TEXAS MEDICAL JOURNAL

DR. F. E. DANIEL,
Founder.

Established July,
1885

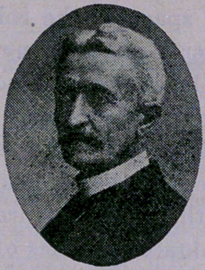

MRS. F. E. DANIEL,
Publisher and Managing
Editor.

VOL. XXXIV No. 10
AUSTIN,
APRIL, 1919.

PUBLISHED MONTHLY.
Subscription:
$1.50

The publisher is not responsible for views of contributors.

ASSISTANT EDITORS:

DR. JOHN S. TURNER, Dallas, Texas.
DR. E. B. PARSONS, Palestine, Texas.
DR. A. P. HOWARD, Houston, Texas.
DR. JOE BECTON, Greenville, Texas.
DR. M. F. BLEDSOE, Port Arthur.
DR. J. H. FLORENCE, Tulsa, Oklahoma.
DR. J. H. REUSS, Cuero, Texas.

### EDITORIAL.

### Looking to the Future.

For the past two or three years, even since the beginning of the European war, medical publications have been largely handicapped in their work. Many of the leading physicians of the country have either been in the service, or if left at home they have been so busy that they could hardly find time to read the medical journals much less to write contributions for them. The manufacturing chemists have been unable to supply the demand for their products, because they could not get supplies, and therefore they have hesitated to advertise, and a number of them have decreased their advertising.

However, conditions are rapidly becoming normal. Most of the doctors are returning home from the service and resuming the practice. They will become readers of and contributors to the medical journals. In fact they will have much more to write about than if they had not gone into the service, and their broadened experience will be such that they will be glad to impart to others the information they have gained.

Then, it has been impossible to increase subscription lists and the journals that have maintained their normal subscription lists have been fortunate. The doctors who are coming back are subscribing for their favorite journals, and there is so much that is new in medicine that every doctor will have to become a large reader of medical literature or become fossilized.

All the progressive firms that cater to the needs of physicians and surgeons are taking space in the .columns of the medical journals. There are several pages of new advertising in this issue of this journal and the correspondence indicates that there will be many more soon. Doctors may be sure that manufacturers who have anything worth while to place on the market are going to advertise it in the medical journals. The firms that do not advertise in these days of progressive business are firms that should be let alone. Articles of merit seek publicity of the proper sort through the right channels. It is generally recognized that the medical journals are the right channels for reaching doctors.

Taking it all in all there is a bright future, now that the war is over, for the doctors of the country, for the wide-awake medical publications and for the enterprising firms that seek to do business with the doctors.

